# Trust and the Goldacre Review: why trusted research environments are not about trust

**DOI:** 10.1136/jme-2022-108435

**Published:** 2022-08-23

**Authors:** Mackenzie Graham, Richard Milne, Paige Fitzsimmons, Mark Sheehan

**Affiliations:** 1 Wellcome Centre for Ethics and Humanities, University of Oxford, Oxford, UK; 2 Wellcome Connecting Science, Wellcome Genome Campus, Hinxton, UK; 3 Kavli Centre for Ethics, Science and the Public, Faculty of Education, University of Cambridge, Cambridge, UK; 4 Ethox Centre, Nuffield Department of Population Health, University of Oxford, Oxford, UK; 5 NIHR Oxford Biomedical Research Centre, Oxford, Oxfordshire, UK

**Keywords:** ethics, health care economics and organizations, information technology, public policy

## Introduction

The significance of big data for driving health research and improvements in patient care is well recognised. Along with these potential benefits, however, come significant challenges, including those concerning the sharing and linkage of health and social care records.

Recently, there has been a shift in attention towards a paradigm of data sharing centred on the ‘trusted research environment’ (TRE). TREs are being widely adopted by the UK’s health data initiatives including Health Data Research UK (HDR UK),^1^ Our Future Health^2^ and Genomics England.^3^ A recent review commissioned by the UK’s Department of Health and Social Care (hereafter referred to as the ‘Goldacre Review’) places TREs at the heart of its recommendations around the future of National Health Service (NHS) health data sharing for research, describing them as the ‘clear path forward’ to a health data system in which trust is ‘earned’ through ‘provable, credible steps to protect patient privacy, and by being transparent with everyone about everything that is done with their deepest medical secrets’.^4^

We argue that rather than building public trust, the TRE model actually *reduces* the need for trust in the use and sharing of patient health data. This is because trust is importantly connected to vulnerability and uncertainty; an essential part of trusting someone is accepting that one’s trust could be disappointed or betrayed. In attempting to provide assurances or guarantees of data privacy and security, TREs strive to remove this vulnerability, and so remove the need for trust. We do not see this as a problem and are broadly supportive of this kind of data sharing model because of the increased security and oversight it provides. However, having argued that TREs are not actually concerned with trust, we consider the importance of being precise about the words that we use in the context of health data sharing.

## What is a trusted research environment?

A TRE is a controlled computing environment that provides remote access to health data for approved researchers via a virtual desktop. Researchers cannot remove individual-level data from the TRE but can export analysis results (eg, aggregate-level results) after approval from data custodians. An analogy is often drawn between TREs and ‘secure reference libraries’, where readers are given access to the books (‘data’) they need for a specific purpose, but the books themselves do not leave the library space.

TREs have been argued to be a desirable alternative to other data distribution models for several reasons.^1 4^ First, the ‘data release model’—where processed datasets are distributed to researchers—increases the risk of reidentification by introducing the possibility of data being accessed by unknown third parties and potentially linked with other datasets. Second, data protection regulations such as the UK’s Data Protection Act 2018^5^ impose severe financial penalties for failing to adequately protect personal health data, which may have resulted in data custodians becoming more risk averse with regard to sharing data. It has also become increasingly challenging for organisations to operate their own computing environments with the level of security required by these regulations. Third, data distribution is both inefficient and costly, particularly for large datasets (eg, medical images and genomic data.) Finally, advances in computing systems have made it more feasible for centralised systems to support complex custom analysis algorithms at the scale required by researchers.

A further putative benefit of the TRE model is its role in building public trust towards the sharing of health data. This is central to the articulation of TREs in the Goldacre Review. By maintaining the security and privacy of health data, TREs are presented as part of an approach to ‘[b]uild trust by taking concrete action on privacy and transparency’,^4^ actively addressing the concerns of the public about data sharing and thereby demonstrating trustworthiness.

## TREs are not about trust

While there is no agreed upon analysis of trust and trustworthiness among philosophers, a few features are widely shared amongst the various accounts. First, trust is an attitude we have towards people that we hope will be trustworthy, while trustworthiness is a property of those in whom trust is well grounded. Second, trust involves expectations about the competence and willingness of the trusted person (and on most accounts, it involves more than this). Because trust involves expectations on the part of the trusting person—expectations which may not be fulfilled—trusting creates vulnerability: without the possibility that our trust might be disappointed or betrayed, there is no need to trust.^6 7^ For example, if I trust a friend to look after my prized orchids while I am on vacation, I make myself vulnerable not only to a potential bad outcome (the death of my orchids), but the betrayal of my trust by my friend.

How we understand the concept of trust has implications for how we approach this vulnerability. On some accounts, trust implies a belief about the trustworthiness of the person being trusted.^8–10^ The more evidence of trustworthiness we have, the more well-grounded our trust in that person. However, once we have decided to trust, further evidence gathering seems to undermine, rather than enhance, trust. Consider the following example: a parent leaves their child with a babysitter to go out for the evening but continues to monitor the babysitter through a series of hidden cameras. Despite the fact that the parent is seemingly broadening their evidence base about the trustworthiness of the babysitter (ie, they are continually updating their beliefs about the competence and willingness of the babysitter with new information), it seems obvious that the parent is failing to trust.

On other accounts of trust, trusting does not necessarily involve a belief about trustworthiness, but rather involves viewing the object of trust as an autonomous person to whom ‘reactive attitudes’, such as gratitude, resentment, and betrayal, are appropriate.^11^ For example, I might *rely* on my phone battery to remain charged for a full day (I act as though it will by not bringing my charger), but I do not *trust* the phone because I do not view it as something to which I would feel grateful it if it stays charged, or betrayed if it does not. Conversely, I might trust a stranger with my phone to take a picture of me and my friends; if they run off with my phone, I would rightly feel betrayed.

The relevance to TREs is readily apparent. The Goldacre Review characterises TREs as providing a means of ‘earning public trust through transparency and accountability’, through ‘technical barriers to [data] misuse… monitoring to ensure all activity remains within the permissions granted, and where all uses are automatically disclosed’.^4^ Yet, imposing barriers to access, continuous monitoring of data use and regular auditing—while possibly effective means of enhancing data security—are not a means to building trust in the health data system. Rather, these methods render trust unnecessary by eliminating the ways in which it might be disappointed or betrayed.

Consider another institution that emphasises the importance of security: banks. If my bank replaces its old vault with a state-of-the-art, high-security vault, my money may be more secure, but not because the bank (or the vault itself) is more trustworthy than before. Similarly, if the vault requires codes from multiple managers to open, my money may be more secure, but not because the managers are more trustworthy. While it may be more difficult for them to betray my trust, this is very different from saying that they, or the bank itself, are more trustworthy. Indeed, I no longer need to trust any single bank manager, because it is no longer *possible* for them to betray my trust by stealing my money on their own.

Of course, as this example illustrates, there is still a need for trust to some degree, insofar as security is not infallible (the two managers might work together to steal money from the vault, for example). Equivalently, in the broader network of health data sharing, there will similarly be a need for trust in various places. However, just as increasing the security features of the vault does not make it more trustworthy, and constraining the freedom of the managers to access the vault does not make the vault or managers more trustworthy, the added security, auditing, and monitoring of a TRE may increase the security of health data, but does not make the TRE itself, or the broader network of health data sharing, more trustworthy.^1^

The language of ‘building public trust’ suggests that the subjects of trust in the context of TREs are primarily the public, so they will be our focus here. However, it is not clear based on the Goldacre Review who or what the object of trust is meant to be: the researchers using the data, those tasked with granting access to it, or the TRE itself. (Nor is it clear, incidentally, who the public are or what it might mean to assess their trust.) Emphasising the data security offered by the TRE suggests that the object of public trust is the TRE itself, while highlighting the transparency and accountability offered by the TRE suggests that the objects of trust are the data users and those that grant access to the data.

Furthermore, it is unclear exactly what it means to ‘build public trust’ in data sharing. Philosophers often describe trust as fundamentally a three-place relation: A trusts B to C (or with C, or in the role of C, etc).^6 12^ Accordingly, ‘public trust in data sharing’ might mean something like ‘the public trusts X institution(s) to use their data in certain specifiable ways for certain specifiable purposes’. While we would need to more clearly define the terms of this relation, it is fundamentally a relation in which the institutions are being trusted to *do* something.

Alternatively, trust is sometimes understood as fundamentally a two-place relation: A trusts B. Here, the trusting person is not envisioning a particular act or role that the trustee will perform. Rather, A trusts B *simpliciter*. This does not require that trust is all or nothing, nor that everyone we trust, we trust in the same way. Rather, when A trusts B, A trusts them in the way that is appropriate given the type of relationship that exists between them.^13^ Thus, what it means for the public to trust the institutions using their data will depend on the nature of the relationship but will not simply be reducible to a list of tasks the institution is trusted to perform (as in a three-place relation).

Suppose that ‘building public trust in data sharing’ is meant as a three-place relation, where what is being trusted is that health data will be stored, shared, and used in a way that protects individual privacy and security, allows research to take place, and benefits the public. First, restricting access to certain datasets and not allowing them to be removed from the TRE prevents trust in potential data users. Specifically, the use of a TRE makes the belief that researchers will use data appropriately redundant; they do not need to be trusted to do so, because there is no possibility of their acting otherwise. Similarly, requiring that researchers be monitored in their use of data strives to eliminate the possibility of data misuse, and thus, to reduce the degree of vulnerability of data users. In doing so, however, it also removes the need for trust.

Second, the means of controlling access to data provided by the TRE reduces the need for trust in those decision makers that grant access to data. These decision makers no longer need to be trusted to judge whether applicants are responsible, rather than simply competent, users of data, or whether applicants have the necessary infrastructure to store and use data safely and securely. Of course, they must still determine whether the use of data is in the public interest, for example, and one could argue that this is a matter of trust, although an area that still needs significant further elaboration. Still, it is difficult to see how providing access to data through a TRE thereby builds trust in decision makers granting access, insofar as it constrains the ways in which data can be used. That is, if TREs reduce the risks associated with decision makers granting access to data, this seems to imply a reduced trust in these decision makers, rather than greater trust.

Third, the assurances regarding data security and internal auditing provided by the TRE diminish the need for trust in the TRE itself. Providing assurances or guarantees of performance is antithetical to trust, because trust requires making oneself vulnerable to the possibility that one’s trust might be let down or betrayed. If one has a guarantee of performance, there is no such risk, and thus, there is no trust. If someone will be compelled to act in a certain way, there is no need to *trust* that they will act in this way. Moreover, continuous monitoring of someone’s activities undermines, rather than builds, trust, because it suggests the lack of a robust belief that the ‘trusted’ person will do what they have been trusted to do. Offering the public transparency and regular auditing of the TRE’s activities (ie, who data is being provided to and for what purposes) enables the public to forego trusting the TRE by striving to eliminate the possibility that the TRE will act in ways other than it has been ‘trusted’ to.

However, suppose that ‘building public trust’ with respect to data sharing is meant as a two-place relation. This suggests that institutions using and sharing health data want the public to treat them as they would someone they genuinely trust and allow them wide discretion to use and share health data in various ways.

However, even if we understand ‘building public trust’ as involving a two-place relation, the use of TREs runs contrary to building this kind of trust in institutions using and sharing health data. Even more so than in an instance of three-place trust, two-place trusting involves making oneself vulnerable to the discretion of the trusted person (or institution). If I trust someone completely, I am willing to give them wide scope over my interests. Conversely, I am willing to provide less discretion, over a narrower range of interests, to those I trust less. By limiting access to health data to certain approved users for certain approved ends, and monitoring and auditing its use, TREs reduce the discretion granted to those using, storing and sharing health data to act (or fail to act) in ways that vindicate or betray trust. While this is a way of reducing my vulnerability to data misuse (insofar as it makes data misuse more difficult), it does not reduce my vulnerability by increasing trustworthiness, but rather by decreasing the discretion allowed to data controllers. As mentioned previously, in attempting to build public trust by increasing security and oversight, TREs render trust not only unnecessary, but reduce its possible scope.

## What’s in a name?

So, contrary to their name, TREs turn out not to involve trust. By providing assurances of data security, enhancing privacy protection and monitoring data use, TREs may make people more willing to allow their data to be used, but in doing so strive to remove the need for trust. Thus, a more appropriate name would be ‘Secure Research Environments’, since this is what they are designed to offer.

We have not argued, however, that these kinds of data sharing platforms are a bad idea. In fact, as a method of enhancing data security and streamlining the conduct of important health research, they appear to have much to recommend them. Moreover, they seem to be responding to what the public appears to want from data sharing platforms. Reports of patient and public attitudes towards the sharing of health data show that the public is broadly supportive of health data sharing for research purposes, provided that data users, and the regulations that govern them, are ‘trustworthy’.^14^ According to these reports, ‘trustworthiness’ depends on a number of factors, including the motivations of those conducting the research, the security measures in place around data access, whether the research is for public benefit, the degree of transparency, and whether data users are held accountable for misuse. In one study cited by the Goldacre Report, TREs were considered by public representatives to be more transparent, more secure, less risky and therefore ‘more trustworthy’ than other kinds of data-sharing initiatives.^4^

Of course, as we have argued, TREs actually reduce the need for trust, precisely because they emphasise factors like providing assurances of performances and reducing vulnerability. Thus, it is a misunderstanding on the part of the public to suggest that initiatives designed to reduce the need for trust are ‘more trustworthy’. Still, if the public has a particular understanding of what is required for a data sharing initiative to be acceptable, and TREs adequately address these concerns, does it really matter if we call these initiatives ‘trusted’ rather than some other term?

We think the answer to this question is ‘Yes’. As described previously, trust invites certain expectations of the trusted person, as well as a readiness to respond in certain ways to the success or failure of trust. Calling something a ‘Trusted Research Environment’ encourages people to ascribe certain features to it, namely, those features that people associate with someone or something they trust. On the one hand, people may not want to make themselves vulnerable with respect to sharing their data in the ways that trust requires, in which case they may not want to ‘trust’ a TRE. On the other hand, and what we think is the more likely scenario, in taking themselves to be ‘trusting’ a TRE, people may expect more of the TRE than it is designed to offer, and reasonably feel betrayed if and when the TRE is not able to offer this. For example, someone might expect that data will be shared only for research that is designed to benefit the public, while at the same time expecting it not to be shared with commercial companies. In this case, an individual’s expectations of the TRE (what the TRE is being ‘trusted’ to do), and the actual purposes of the TRE, are not in alignment.

However, the issue is not simply that there may be disagreement about what the TRE is actually being trusted to do; this could potentially be resolved by being explicit about the role of the TRE in the sharing of health data. The issue is also that being *trusted* to do something is different than being relied on or depended on, and the kinds of reasons for acting that we expect the trusted person to have are correspondingly different. Trust is not merely a rational prediction about performance, and so requires reasons for trusting that go beyond the kind of evidence that would justify a rational prediction. This is illustrated by the earlier example of the babysitter: the cautious parent has plenty of evidence to ground a prediction that the babysitter will take good care of the child, yet they are nevertheless unable to trust.

Perhaps we could argue that TREs are striving to be ‘trustworthy’ rather than ‘trusted’. However, this does not address the fact that ‘building public trust’ is concerned with being trusted, rather than being trustworthy. While being trustworthy is often an effective way of getting others to trust, this is not always the case.^7^ Indeed, I might correctly believe that someone is trustworthy, but choose not to trust them (eg, I don’t want to burden them). Moreover, security, transparency and accountability do not serve to make TREs more trustworthy users or sharers of data; rather, they provide evidence of the ways that trust is not needed.

The bottom line here is that the words that we use make a difference to how people understand institutions like TREs. A trust relationship is a very distinctive one, requiring particular kinds of investments, both emotional and psychological, on the part of the trustor and the trusted. If we say it is a matter of trust, then people relate to the institution differently: their expectations will adjust to what it is to trust and what is expected from the trust relationship. There are, of course, marketing advantages for the institution in calling itself ‘trusted’: ‘trust’ is warm and friendly in a way that ‘secure’ is not. These connotations make a difference to how the institutions are presented and framed to the public. The name matters.

## Moving beyond trust

Building ‘public trust’ has been a major part of the rhetoric surrounding health data sharing. With the burgeoning implementation of TREs, this language is becoming embedded in the structures of health data sharing itself; the way we govern the use of data is influenced by the way we understand the concepts of trust and trustworthiness.

Accordingly, we need at the very least to think carefully about where trust and trustworthiness are appropriate and desirable in the people, institutions, and structures (eg, data platforms) involved with data sharing. This requires being clear about what we mean by ‘trust’ and ‘trustworthiness’, whether this is at the policy level, or when we are surveying public attitudes about issues like health data sharing. Apart from anything else, the public (or publics) are an infinitely complex set of attitudes and behaviours made up of networks of individuals that are influenced by their own experiences and relationships, as well as what they hear and how it is presented. Just as we need to think carefully about what we call our institutions, we also need to be very careful about how we access ‘the public’ and how we present and frame the questions that we ask of them.

It may even be that we need to move beyond the language of ‘building public trust’ in the context of health data sharing. As we have argued, trust is essentially about making ourselves vulnerable to others in certain respects; it is about placing a degree of power over our interests in the hands of another. In many situations, depending on others in this way is necessary and useful. However, not every situation in which we rely on the actions of others demands trust, specifically the openness to the possibility of betrayal that is characteristic of trust. Indeed, it seems reasonable to want assurances or guarantees about how health data is being stored, shared, and used, suggesting that data sharing should not be a matter of trust. When there is no viable means of providing researchers secure access to health data from a centralised database, and the use of health data cannot be monitored directly, there may be a need to trust. If and when data sharing models fulfil the promise set out in the Goldacre Review, this may no longer be the case. What it is reasonable to expect from those sharing, storing, and using health data may change, as well as the degree of vulnerability that the public is willing to accept with respect to their health data.

Accordingly, we emphasise the importance of good governance structures and regulation for institutions involved in the broad network of health data sharing, to promote data security in areas where security is appropriate and to build trustworthiness in areas where trust is appropriate. These structures might include external constraints on the storage, sharing and use of health data (eg, reporting and transparency requirements, appropriate disclosure, prospective review and other oversight, external auditing), as well as internal mechanisms to ensure that institutions take seriously their responsibilities to promote the ethical use of health data (eg, explicit statement of institutional goals and ethos, openness and transparency with respect to decision-making processes, and inclusion of the public or independent advisors in appropriate roles). Part of what make TREs attractive are the ways that they can be used to implement these governance structures. However, at the point that data use is being continually monitored and audited, and assurances of its security provided, we have moved on from trust. At the same time, where we continue to depend on the discretion of others with respect to our health data, there may continue to be a need for trust.

## Data Availability

There are no data in this work.

## Footnotes

We thank an anonymous reviewer for pushing us to clarify this point.
